# This Is My Story (TIMS), a Five-Year Thematic Analysis of Patient Narratives from a Novel Intervention in an Academic Hospital

**DOI:** 10.3390/healthcare14182994

**Published:** 2026-09-14

**Authors:** Jason Wilson, Bikram Bains, Sampath Rapuri, Edgar Robitaille, Catherine Chan, Naomi Crane, Ellen Fei, Elizabeth Tracey

**Affiliations:** 1 Division of Spiritual Care and Chaplaincy, Johns Hopkins Hospital, Baltimore, MD 21287, USA; 2 Department of Biomedical Engineering, Johns Hopkins University, Baltimore, MD 21218, USA; 3 Department of Public Health Studies, Johns Hopkins University, Baltimore, MD 21218, USA; 4 Department of Krieger School of Arts and Sciences, Johns Hopkins University, Baltimore, MD 21218, USA; 5 Department of Molecular and Cell Biology, Johns Hopkins University, Baltimore, MD 21218, USA; 6 Department of Medicine, School of Medicine, Johns Hopkins University, Baltimore, MD 21205, USA

**Keywords:** chaplain interview, narrative empathy, patient narrative, patient perspectives, patient narratives, patient-centered care

## Abstract

**Background/Objective:** This is My Story (TIMS) is a patient-centered, interview-based audio narrative program that provides a humanistic intervention to help the medical team understand more information about a patient’s story that is not routinely captured in their medical chart. The goal was to analyze themes present in the collected TIMS interview narratives and determine how they correspond to the specific interview prompts and discover whether there are any broader patterns and categories in responses. **Methods:** This is a secondary research study using retrospectively collected audio interviews, with hospitalized patients or their legally authorized representative, who provided their oral consent for the recording and future use of these interviews. A total of 362 TIMS interviews make up a subset of data for the reflexive thematic analysis, in which researchers analyzed, with some AI-assisted analysis, the themes that were present when the patient answered four structured interview questions. **Results:** The most common themes found were Experiences and Quality of Care, Family and Social Connections, Peace and Well-being, Joy and Meaning in Life, and Preferences and Personal Identity. **Conclusions:** The themes discovered in response to the four structured questions were directly related and influenced by the questions in which they correspond to, but there is also overlap between the questions, suggesting that each contributed to an overall cohesive patient narrative.

## 1. Introduction

This is My Story (TIMS) is a narrative-based interview that is recorded, edited, and uploaded to the electronic health record (EHR). This interview-based approach has been used in various settings at The Johns Hopkins Hospital, such as intensive care units (ICUs), general medical-surgical units, and pre-operative units. It is routinely conducted with the patient themselves or, if they are unable to communicate, with a loved one who can answer on their behalf. The program began during the COVID-19 pandemic in early April of 2020, when the unit chaplain observed a disconnect between medical providers, patients, and families, as clinician distress was on the rise. The Journal of the American Medical Association [1] identified the inability to maintain a normal human connection with patients or peers as one of the four major factors contributing to moral distress among physicians at the onset of the pandemic. Moral distress, defined as the inability to act according to one’s core values, prevents physicians from acting to address patient needs, ultimately affecting the plan of care [2]. In this context, physician moral distress is not separate from but becomes entangled with patient suffering. Social disconnect has led patients and family members to experience feelings of isolation, demoralization, helplessness, anxiety, and confusion, therefore resulting in heightened emotional distress and perceptions of lower quality of care [3].

The interview is structured and consists of four questions: how do you want to be addressed (Q1); what brings you joy (Q2); what does the medical team need to know to care for you best (Q3); and what brings you peace (Q4). The interviews are edited down to 1–2 min and uploaded to the EHR to facilitate clinician use. Follow-up questions may be asked by the interviewer for more details or clarity. While responses are closely linked to the topic of the question posed, the interviewee is encouraged to answer however they feel most genuinely expresses themselves. It is the sub-themes of response to each question that are of interest to this study. For example, when asking what brings the patient joy, understanding that family, activities and helping others are the common responses (Appendix A) provides context for what clinicians can focus on during their conversations with the patient and families.

The overall goal of this audio intervention is to assist clinicians in seeing hospitalized individuals as people, not just a collection of symptoms or quantifiable data that appears in a medical chart. Failure to do so could potentially result in patients losing trust in their health care provider and refraining from voicing their concerns [4]. Additionally, the information offered by the patient or their loved one may provide actionable details that providers can use in their care or conversations with the person. One study conducted on patient-centered decision-making and health care outcomes found that when physicians considered the context, needs, and circumstances of their patients when planning their care, individualized health care outcomes improved [5]. For instance, addressing a patient’s financial constraints to afford medication led to improved diabetes control, and addressing a patient’s social burden of competing caregiving responsibilities for a chronically ill family member led to fewer urgent care visits. These findings attest to the power of communication and storytelling in daily medical practice. Additional studies demonstrate that narrative competence offers additional benefits for the patient, both in telling their story and in being heard [6]. By engaging with patients through attentive listening and dedicating time to understand patient stories, clinicians may begin to appreciate facets of illness beyond what is captured in the medical chart, such as the social contexts and stigmas that surround illness. By implementing the audio intervention detailed in this paper, we aim to foster a deeper understanding of health and illness by situating each patient within the context of their lived experiences.

This differs from previous studies, as this study does not look at the feasibility, workflow acceptability or potential clinical impact on providers of hearing such narratives. Rather, it provides a thematic analysis of the interviews themselves, where the focus is on the composition of the narratives, which has not yet been systematically studied. This study is the first, in this program, considering the collected interviews of patients, or their loved ones, to see what types of things were said, not just how what patients say might be collected and used in clinical care.

## 2. Materials and Methods

The dataset used is a mixed corpus of 362 audio files acquired from This Is My Story (TIMS) interviews conducted from 2020 to 2024 in the Medical Intensive Care Unit (MICU) and Neurosciences Critical Care Unit (NCCU) at The Johns Hopkins Hospital. Attending physicians and research chaplains identified appropriate candidates for the initial interviews, and we utilized a retrospective sample subset of these recordings. The inclusion criteria for the initial recordings were that the patient, or LAR, provided consent and the patient was expected to be hospitalized for longer than 3 days; this allowed us to edit the interview and upload it to the patient’s electronic medical record. A post analysis of participant demographics, compared to the unit in which these interviews were conducted, revealed a representative sample from which age, identified gender and race were obtained for the selected TIMS interviews. In cases where patients were unable to communicate, interviews were conducted with legally authorized representatives (LARs). Both patient interviews and interviews conducted with an LAR were analyzed together and represent a mixed corpus of direct patient and surrogate narratives. Differences between these two groups are not a focus of this analysis and are considered a limitation to be explored in the future, as such interviewer categorization was not reliably tracked for sub-group analysis. Interviews averaged two minutes in duration and were conducted either in person or remotely over the phone.

AI-assisted coding was used to facilitate the analysis of the large corpus of structured interviews but still maintain the human interpretation of themes. The LLM reduced the burden of creating the suggested coding structure for a first pass rather than determining the final thematic framework. We recognized that LLM-generated codes may reflect biases in model training data, preferentially identify common or linguistically salient concepts, overlook uncommon experiences, or influence subsequent human interpretation through anchoring. Because of this, the LLM outputs were only treated as suggested preliminary themes.

### 2.1. Qualitative Analysis

Audio files were transcribed using Nvidia’s parakeet-tdt-0.6b-v2 automatic speech recognition model. The team did not report any major errors in ASR that prevented them from understanding the transcript. The analysis was inductive and used a hybrid approach informed by reflexive thematic analysis and template analysis, with AI-generated preliminary codes serving as the initial scaffold that was iteratively refined through human coder review, consensus discussion, and special attention to emergent themes. We used OpenAI’s GPT-4o model, an LLM hosted on Johns Hopkins private servers which is HIPAA-compliant, to ingest the transcribed interviews and generate a preliminary set of codes in two batches due to context limits. The main system prompts are included in Appendix A with additional codebook prompts available in Supplementary Materials. No codes were generated prior to the use of AI, as they were derived from the transcripts themselves. Code granularity was refined by the human coders after reviewing the suggested preliminary codes from the use of the LLM. The initial codes were treated as a scaffold to understand what could potentially be represented in the dataset but need to be organized more organically to accurately reflect the narratives’ intent and how they were being understood for analysis. The transcripts were randomly validated by listening to the audio file and then verifying that the audio had been accurately captured. This is reflected in the subset of audio files used; initially, 749 were submitted for transcription, and only 362 were found to be reliably transcribed without a probable error in the transcription process. Files were assessed by human code reviewers for reliability in 2 categories: (1) there was a duplicate of another file in the dataset, and (2) all four questions had a response that appeared to be loosely related to the prompt which could be understood audibly. Response applicability to the question was generous and only questioned when it was very evident that this was not an answer to the prompt but rather extraneous audio captured during the interview. There was a 50% threshold that was maintained; if more than 2 of the questions had a concern, in terms of applicable content or audibility, it was removed. During the interview process, the interviewer did not discourage specific responses to the prompt and progressed through the interview questions regardless of the response. Meanwhile, in the process of reviewing the transcripts, it was noted that some questions were skipped or non-audible. Duplicate audio files were highlighted during the AI transcription process as potential duplicates, and once human reviewer confirmation was conducted, they were deleted from the dataset. The files that were excluded from the dataset were not systematically different from the ones that were retained; they were either incomplete in terms of the 50% threshold or a duplicate of another file. Concerns with the audio files were mostly found in the first 2 years of the program, and this was attributed to early inexperience with capturing these interviews reliably and record storage as many were duplicates. During the initial phase of this study, during the period of COVID-19 precautions being taken and interviews being conducted over the telephone, it was common to pick up on audio during an interview that was not related to the prompt. De-identification was handled by an AI prompt that was used to remove names from the transcripts. These audio files do not have anything in them beyond the first name of patients.

The AI-generated codes were treated as preliminary analytic suggestions rather than definitive interpretations of the data. Following independent review, members of the coding team convened when necessary to discuss differences in interpretation, incorporating emerging themes and proposed changes to the developing coding framework. When reviewing the LLM preliminary codes, changes to the code themselves were not often necessary, but larger groupings were made, combining many smaller category representations. For example, the LLM may have created three distinct categories for narratives discussing walking with a dog, walking with one’s family and walking outside. The coding team would have grouped them together into a larger code of activities that are accounted for in Q2. Five qualitative coders with diverse backgrounds ranging from engineering to chaplaincy manually reviewed, refined, and augmented the GPT-4o outputs [7]. Through an iterative process, the team met to discuss emergent themes, consolidate redundant categories, and refine theme definitions until reaching consensus on a finalized set of themes. Before the consensus meetings, the coders independently reviewed AI-generated codes and reviewed individual quotations to verify the categorization. During group discussions, researchers considered how disciplinary background and familiarity with the intervention could shape the interpretation of the narratives and returned to the source transcripts when differing perspectives affected coding decisions. Disagreement resolution, for the larger grouping of code attribution, was handled by each reviewer re-reviewing the quote in question and hearing the perspective of each of the team members, and then consensus was achieved for the final theme attribution. Often, codes did not change in terms of their larger categories but were seen as being represented in multiple themes.

Following the completion of the hybrid thematic analysis, we observed that the themes found from exploratory analysis appeared to organize into two broader patterns across the dataset. These patterns were not predefined but emerged through a comparison of themes and reflection on their relationships to one another. We refer to these as the external and internal domains, with the external domain capturing patients’ descriptions of events, relationships, and circumstances and the internal domain capturing patients’ interpretations, emotional responses, coping, and meaning-making.

### 2.2. Quantitative Analysis

In this qualitative analysis, frequency counts are presented only as descriptive indicators of thematic emphasis to contextualize the narrative findings, rather than as primary quantitative outcomes. Using these finalized themes, we mapped each one back to the TIMS interview question it was in response to, to understand how the distribution of themes varies across each question. After each patient’s responses were codified and organized under a theme, the frequency of each theme was measured using the total number of codes following a question across all interviews as a denominator. To characterize the thematic composition of an average TIMS interview, the total number of coded instances assigned to each theme was divided by the 362 analyzed interviews. These theme-specific averages, used as contextual markers, were then normalized to represent their proportional contribution to the average interview composition. We additionally studied temporal trends in each theme, as an exploratory outcome, correlating trends across time with major changes in the world and within The Johns Hopkins Hospital (such as the COVID-19 pandemic). During data collection, this was not specifically documented for future analysis, but we will attempt to see whether there are any visible trends based upon the calendar year that the interview was recorded. 

## 3. Results

**Figure 1:**
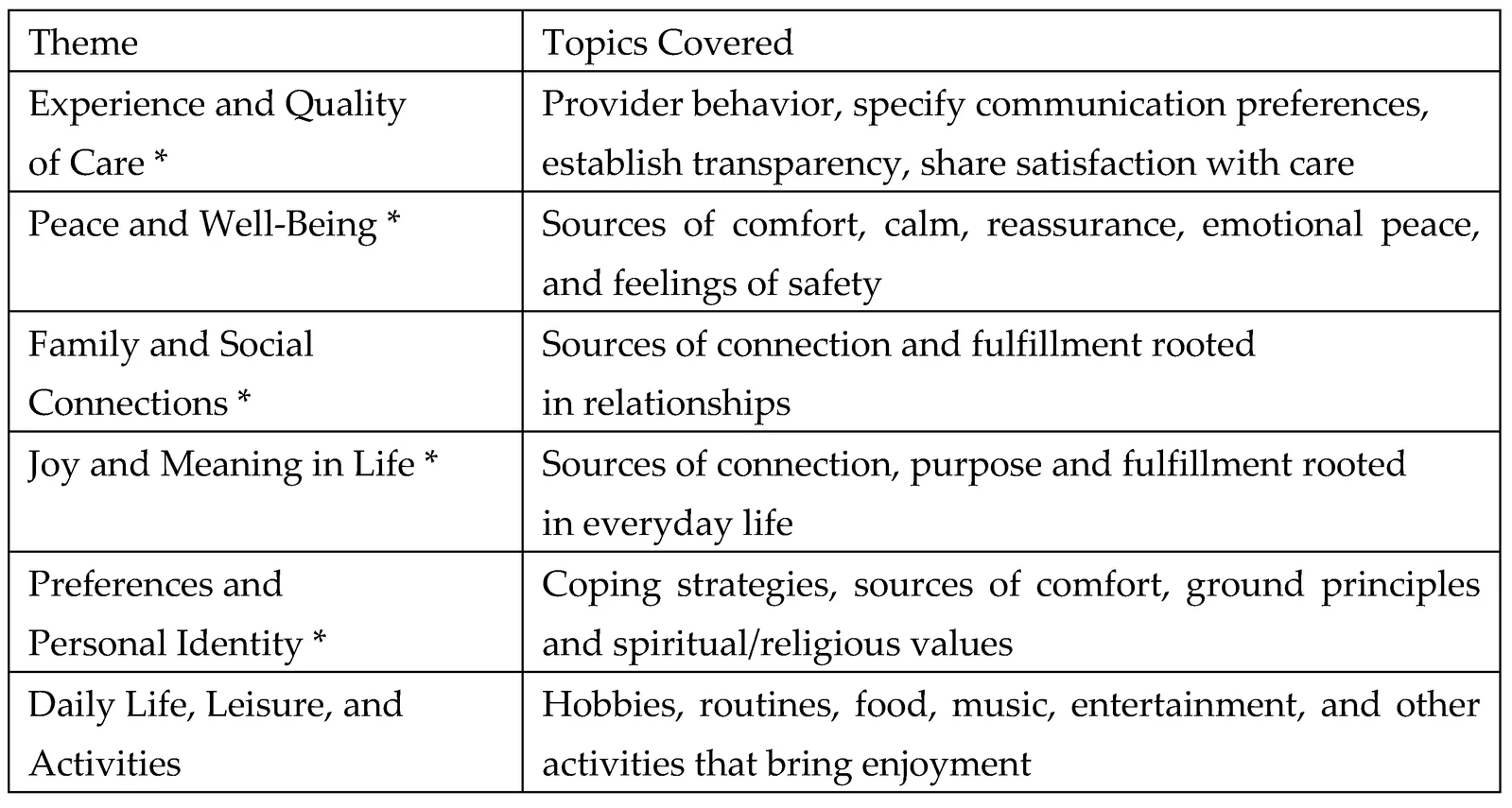

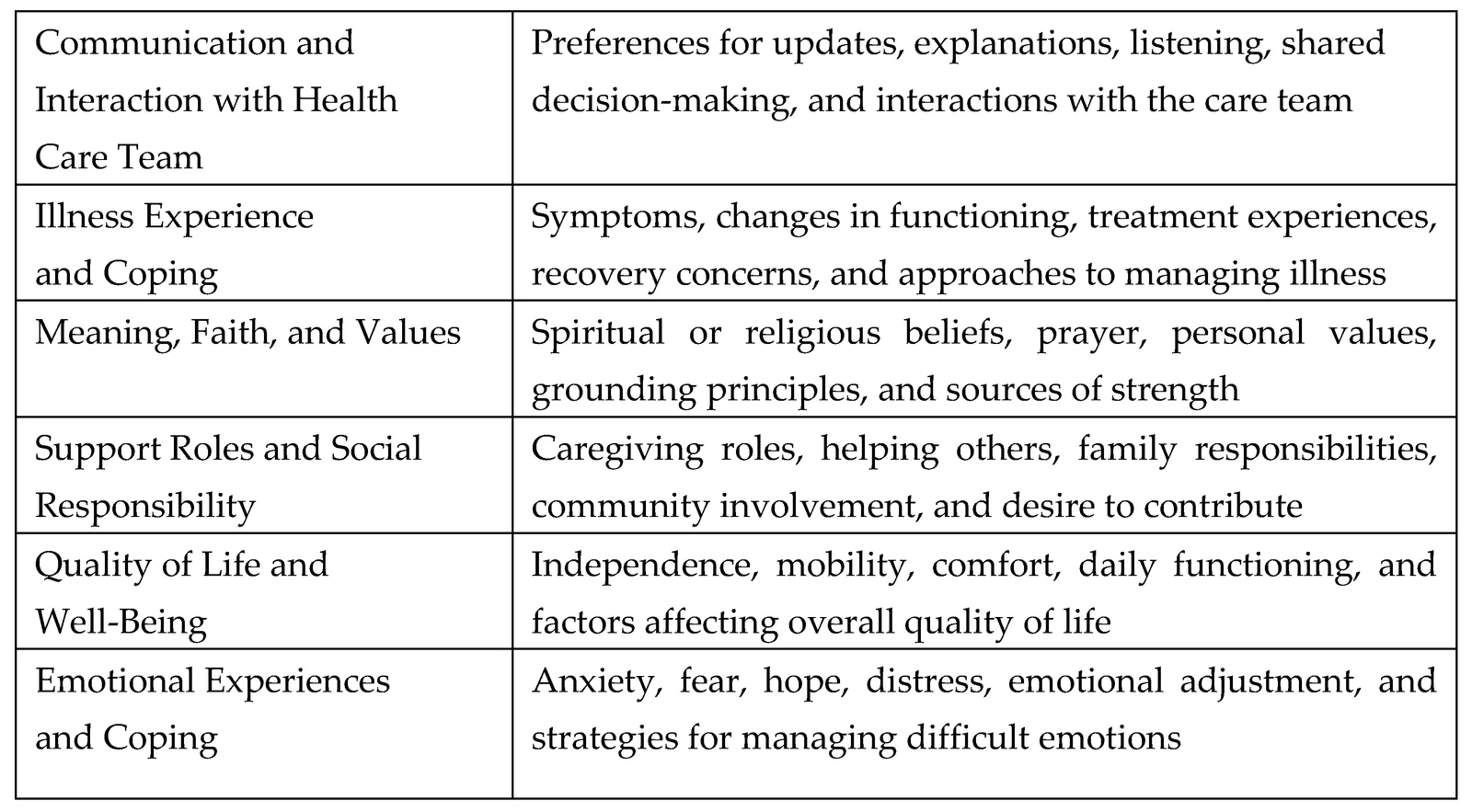
Twelve identified themes. Each theme is identified and common category characteristics listed. Additional thematic attribution is in the Supplementary Materials. * Denotes one of the five frequently occurring themes.

In our analysis, we identified twelve overarching themes, with the five most frequently occurring themes highlighted with asterisks in Figure 1. Specifically, these primary themes consisted of Experience and Quality of Care (most associated with Q3—knowledge of patient), Family and Social Connections (most associated with Q2—joy), Peace and Well-being (most associated with Q4—peace), Joy and Meaning in Life (most associated with Q2), and Preferences and Personal Identity (most associated with Q1—addressed). While most of these themes were present to some extent across many of the four interview questions, each mapped predominantly onto a specific question with additional code re-identification and attributing. There was no distinction made for interviews conducted with the patient or their LAR, as datasets were transcribed and initially analyzed without any note of the source.

**Figure 2:**
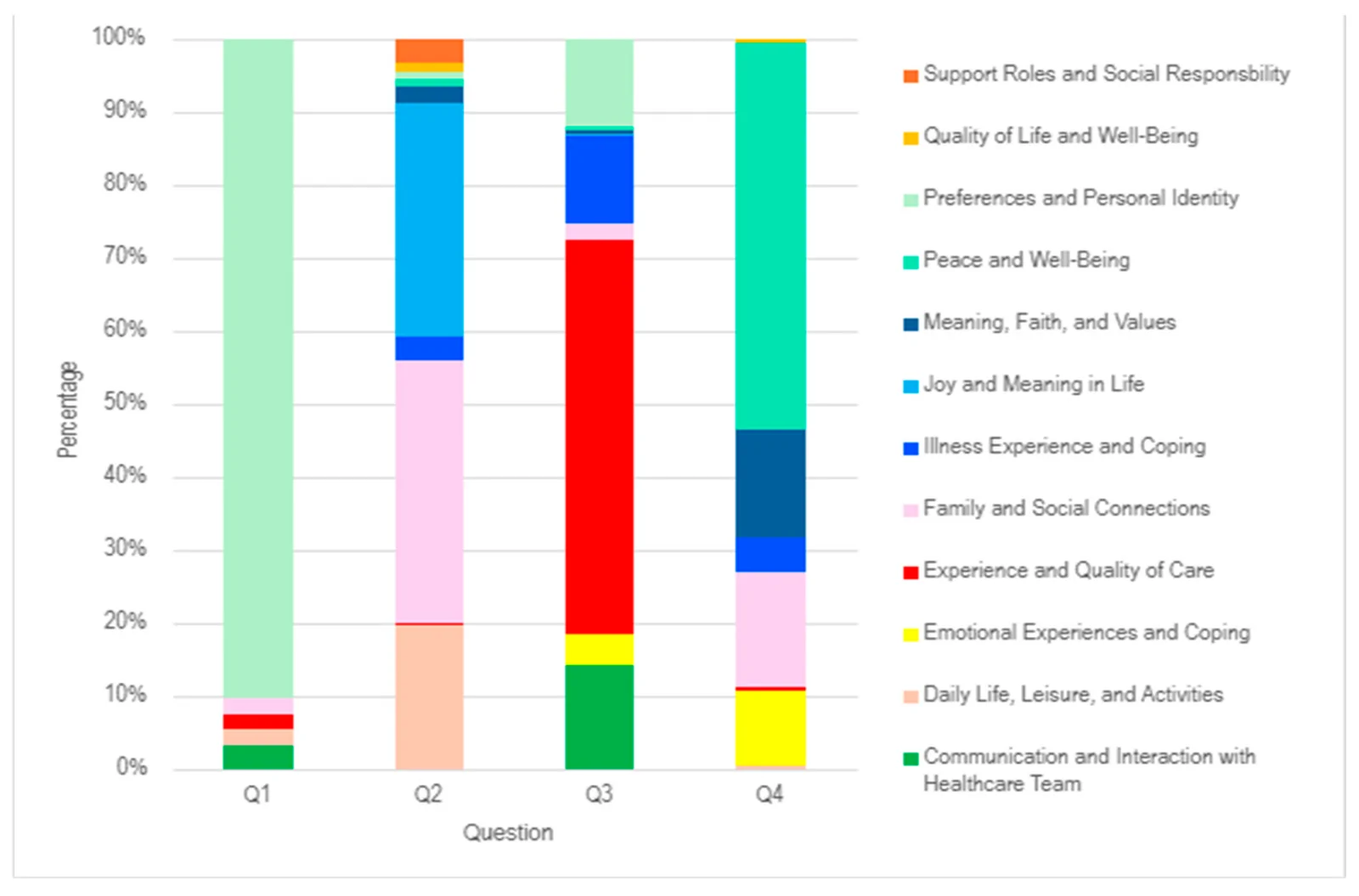
Distribution of thematic content across interview questions. Vertical 100% stacked bar chart showing proportional distribution (by occurrence of theme/sub-themes) of coded themes across four interview questions out of 362 interviews.

Themes related to the health care system, such as “Experience and Quality of Care,” emerged the most frequently and were most often assigned to Q3 responses. On average, this theme appeared in more than half of the interviews and was the most dominant across the dataset. Q3 asks interviewees what the medical team needs to know about the patient to provide the best care. This space was often used to comment on provider behavior, specify communication preferences, establish transparency, and share satisfaction with care. Notably, elements of the care experience appeared even when Q3 was not directly prompted. One patient stated the following: “Just working with me and letting me know the situation and so that I can fully understand what’s happening…I have anxiety, so I always am like not sure what’s happening.” Another patient described the following: “I think they know pretty well. This team down here is the best team, I think, in the hospital. I got put in the wrong room before, and they have no clue. I’ve been here a couple of times, and they’ve been great” (Figure 2).

In contrast, themes related to relationships with family, social connections, and daily life appeared the most in response to Q2. “Family and Social Connections” emerged as the second most frequent theme, appearing in roughly half of all interviews. Similarly, “Joy and Meaning in Life” was assigned to interviewees’ responses to Q2. One patient, in response to Q2, explained the following: “My family, my wife, playing ball, playing softball. I play all over the country or used to. Friends, boating, which I can’t do anymore.” Another patient who solely brought up familial relationships as what sparked joy in them stated the following: “What brings me joy is my family, being around my family, enjoying their birthdays, their get-togethers. Everything that we do is family-centric.” On the contrary, a patient who did not explicitly include familial relationships in their response but included modes of leisure alone stated the following: “A lot of stuff. Music. Movies.”

Themes related to emotional and inner reflection were the most strongly associated with Q4, which asks patients to consider sources of peace. As anticipated, “Peace and Well-being” mapped onto Q4 responses more so than any of the others. This theme appeared in less than half of all interviews, making it the third most common theme overall. Interviewees often used this question to share coping strategies, sources of comfort, grounding principles, and spiritual values. Many patients brought up Christian values, explaining how their faith in God brings them peace, as one patient noted the following: “God. Jesus. He brings me a lot of peace. When I get stressed, he calms me down. Like the other day, I gave them all a scare, even scared myself. They sent me for a MRI with contrast, and I must have reacted. I got terribly cold and started shivering. All of a sudden, all the doctors and nurses were descending upon my room. It was scary. But I just called on Jesus.” Patients also often shared coping mechanisms, as another patient described the following: “Music, any kind of music. I love music. I love music in the ocean, any water, matter of fact. I listen to it every night before we go to bed, that’s how we go to sleep”.

Themes related to one’s identity were the most associated with Q1 responses; however, they were less frequent overall. Among those dealing with identity, the theme “Preferences and Personal Identity” was most frequently assigned to Q1 responses and appeared in approximately one-third of all the interviews. This aligns conceptually with Q1, which is intended to establish how a patient wishes to be addressed. Nevertheless, compared to other themes, identity-related responses were more variable in both frequency and their correspondence to a given question. A poignant example of this was provided when a patient shared, “I prefer to be addressed the right way, you know.” This often was a name, or a name of their loved one if a family member was being interviewed; however it also included a title such as doctor, mister, and reverend.

**Figure 3:**
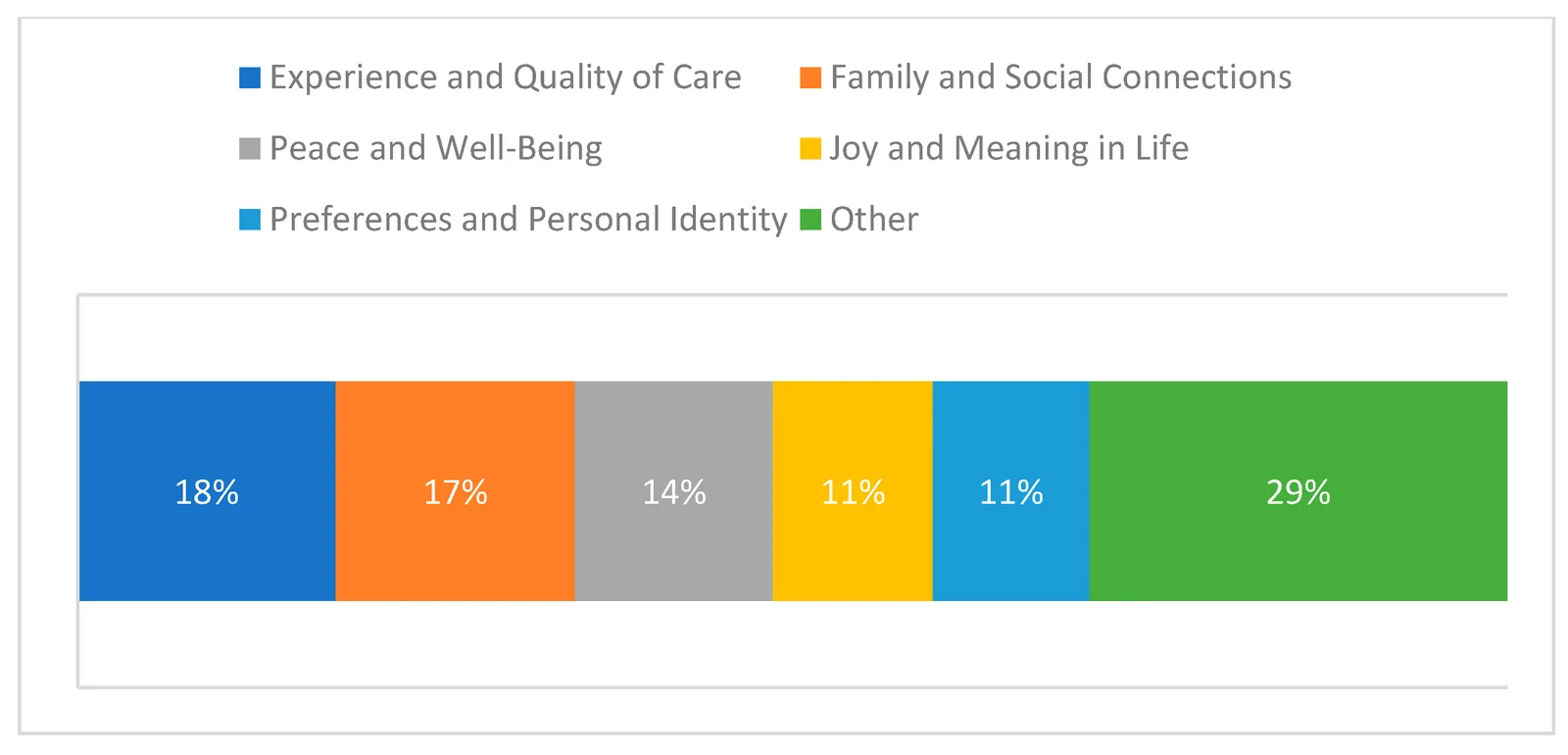
Average interview composition by thematic category. Horizontal 100% stacked bar chart showing average proportion (by occurrence of theme/sub-themes out of 362 interviews) of interview content assigned to each thematic category. Each colored segment represents distinct theme, with length representing relative contribution to average interview.

Twelve themes were identified across the 362 analyzed audio files. As shown in Figure 3, the average interview composition consisted predominantly of five themes that appeared consistently. The dominant themes (Experience and Quality of Care, Family and Social Connections, Peace and Well-Being, Joy and Meaning in Life, and Preferences and Personal Identity) accounted for a little less than three-quarters of the interview’s thematic content. Thematic content was counted when the participant provided a response and the AI provided a code that eventually corresponded to a theme identified by the reviewers. The remaining other seven themes, as outlined in Figure 1, account for a little less than a third of the interview content, and while less prevalent, they provided context and nuance and contributed to the cohesiveness of the patient stories. They are not differentiated across each of the seven themes as their individual contribution was not the focus of the interview composition.

## 4. Discussion

The primary goal of this study was to discover and characterize themes in the TIMS narratives while examining how they are distributed across the questions that elicited them. The findings showed that the four question prompts provided distinct but overlapping themes. The most common themes found were Experiences and Quality of Care, Family and Social Connections, Peace and Well-being, Joy and Meaning in Life, and Preferences and Personal Identity, which make up almost three-fourths of the interview’s content, illustrating the thematic salience of the five main themes discovered. These results suggest that the TIMS conversations consistently elicited a small set of recurring thematic domains that were associated with specific interview questions. It was expected that the overall themes would be reflective of the questions that evoked the response; however we intended to learn what sub-themes appear in the responses. For example, as discussed in Section 3, asking how someone wants to be addressed most often resulted in them giving their name, but sometimes it was an invitation for them to provide their professional credentials or what they liked to do.

In addition to the identification of individual themes, we also unexpectedly observed two broader domain patterns in our exploratory ad hoc analysis from the thematic distributions. Namely, themes generally capture two categories of information—external and internal. Themes that fall into the external domain are associated with the information that an interviewee shares with their interviewer (i.e., what has happened or is happening to a patient; their life experiences). Other themes convey information about how each patient processes or reflects on those experiences. We assign these themes to the internal domain. Thus, themes in the external domain communicate events, relationships, and circumstances that patients describe happening to or around them, while internal domains tell us more about how patients interpret and make meaning of those experiences through emotion, coping strategies and self-care, and values.

The distribution of themes in both domains across interview questions suggests that this external/internal experience distinction may exist on a spectrum or continuum. Many themes certainly communicated a mixture of both internal and external perspectives. While themes in the internal domain are evoked more explicitly by the questions asked during the interview, patients often elaborate on and justify those experiences with information that would fall into the external domain of information. The theme of Experience and Quality of Care would fall into the external domain and was the most strongly associated with Question 3, which asks what the medical team needs to know to care for the patient the best. Family and Social Connections would also fit into the external domain and was the most strongly associated with Question 2, which prompts patients to share what brings them joy, perhaps by reflecting on the role of relationships and their social context. The last three themes—Joy and Meaning in Life (strongest association with Question 2), Peace and Well-being (strongest association with Question 4), and Preferences and Personal Identity (strongest associations with Questions 1 and 3)—primarily reflected the internal domain, capturing how patients interpret their experiences, maintain self-integrity, and find meaning and value in the face of illness.

1. What does the medical team need to know about you to care for you best?

Many responses emphasized the desire for clarity and transparency in communication between medical professionals and patients, regarding this aspect as a highly contributing factor in determining the quality of care. Other responses to Q3 also involved expressing satisfaction with care and comparing it with prior hospital experiences. Responses to Q3 provide beneficial feedback that medical staff can utilize to assess the care they provide and discover potential areas of improvement. By understanding a patient’s previous hospital experiences, staff are better equipped to approach specific procedures and situations to provide the best quality of care tailored to the patient. Notably, elements of the care experience appeared even when Q3 was not directly prompted. This observation suggests that perceptions of care play a role in how patients frame their hospital experiences more broadly. This aligns with Browne’s [8] finding that “patients place much value on effective communication with their providers, the responsiveness of clinicians and staff to their needs, and an overall sense of being treated with care and respect.” This baseline of previous experience was found to directly affect “patients’ engagement with and adherence to providers’ instructions, and clinical processes and outcomes”.

2. What brings you joy?

The Family and Social Connections and Joy and Meaning in Life themes were the most associated with Q2 responses, with both themes often integrated into a singular response. Because Q2 asks interviewees to consider what brings the patient joy, it likely prompts them to reflect on sources of connection, purpose, and fulfillment rooted in familial relationships and everyday life. Chochinov [9] found a similar grouping in their study on dignity therapy with dying patients, where there was a connection found between family relationships, living everyday life and how this resulted in providing meaning in one’s life. The responses to Q2 provide context that will be important for understanding a patient beyond their immediate medical needs and help to humanize their care and treatment.

A common pattern in Q2 responses was the mentioning of “family” and “friends” as what brings the patient joy, which directly correlates with both themes. The inclusion of sports and leisure activities also often appeared in Q2 responses, which closely aligns with the “Joy and Meaning in Life” theme and indicates the integral role of activities in shaping life fulfillment. However, patients also gave responses pertaining to only one of the two themes. The variability in response length and content suggests the multifaceted nature of how joy may transpire and be reflected upon.

3. What brings you peace?

The Peace and Well-being theme was the most associated with Q4 responses, where patients shared their sources of peace. It is clinically beneficial for medical personnel to be informed of a patient’s source of peace, as they can aid the patient to begin to experience a state of peace by initiating simple actions, such as placing a bible by the bedside table or turning on calming ocean wave sounds.

This question specifically allowed the visit with the patient to continue as a chaplain visit, consisting of spiritual care, when it elicited a spiritual or religious value. It was used as a segue into a deeper discussion with the patient, if they were open to it, and the interview itself gave the chaplain the ability to explore many of the concepts that have been discussed when responding to the four interview questions. In contrast to the other questions, Q4 appeared to elicit more reflective, inward-facing responses. These responses suggest that Q4 prompted interviewees to contend with their own considerations of meaning and acceptance. The use of peace in a patient interaction is a common practice, specifically in spiritual care, but can be used by any provider once it has been identified. This model of biopsychosocial care is consistent with other research [10] and our findings in this analysis.

4. How do you want to be addressed?

The Preferences and Personal Identity theme was the most associated with Q1 responses, where the patient detailed how they wanted the interviewer to address them. This question had the most observable variation in the frequency and category of response. This variability may in part be attributed to inconsistencies in how Q1 was asked or interpreted. It may also be driven by the broader scope of identity-related themes, which could reasonably be assigned to any of the four questions. It was discovered that the variability was due to a fundamental misunderstanding of whether the patient wanted to be addressed by a name, given or declared, or rather a title that they wanted the medical team to be aware of in providing care. This misunderstanding or confusion of what is being asked of in this question is found often in LGBTQ+ medical care as the first name is gendered and carries more connotations that just being a way to address a person [11]. As found in this research, this is a gateway to appropriately collaborating with the patient. Developing rapport with a patient could even extend to trust in the relationship, beginning in the first moments of addressing them by their preferred name and title. Often in moments of delirium, as a medicine-related side effect or condition-specific symptom, using a name that the patient preferred seemed to elicit a quicker reorientation of the patient anecdotally.

5. Variability in question administration

In the potential administration deviations, it was noted that some interviewers shared all four questions before responses were provided. In such cases, it becomes more difficult to map responses onto each of the four prompts during coding. In more conversational interviews, participants often shared additional details not directly related to their preferred name. In such cases, we observed themes that might not otherwise be expected to emerge in responses to Q1. Each of these cases is described in detail below.

In some interviews, the first question asked differed from the standardized Q1, eliciting codes other than “PREFFERRED_NAME” and its variants. For example, one interview opened with the question, “Mind just introducing yourself for the recording?” The nature of this question is more open-ended compared to the standardized Q1, and the act of introducing oneself implies providing information beyond one’s name. Therefore, codes such as “LOVES_MUSIC,” “LOVES_FAMILY,” and “JOB_DESCRIPTION” were unexpectedly derived from the Q1 response and mapped to themes unassociated with personal identity.

In another case, some codes categorized as mapping to Q1 corresponded to a preliminary question before standardized Q1. For instance, in some interviews conducted with the patient’s spokesperson, who was often a family member, instead of the patient themself, the interview would first ask the following question: “Who are you and how are you related?” The code “IDENTITY_AND_RELATION” was derived from an interview pertaining to this scenario and mapped to the theme “Family and Social Connections” in addition to “Preferences and Personal Identity.” In a similar instance, the interviewee provided additional information prior to any question being asked, also prompting codes not pertaining to identity to have emerged.

The last case observed was when the standardized Q1 was the first question asked. However, additional codes mapping to themes separate from “Preferences and Personal Identity” were still obtained because interviewees instinctively elaborated upon their name and or identity. The code “PROFESSIONAL_BACKGROUND” derived from a response to the standardized Q1 is one example of this case because the patient preferred to be addressed with their doctoral title, so the interviewee spoke about the patient’s career after stating how they would like to be addressed. The variation in response length and depth to the standardized Q1 may be attributed to how the interviewer conducted the interview. Some interviewers preferred to share all four questions with the interviewee before the formal start of the interview. In those interviews, the codes obtained may have appeared to map more closely to the most frequent theme, corresponding to the question number. On the contrary, other interviewers preferred to immediately start the interview without providing an overview of the questions to the interviewee. As a result, interviewees may have been more inclined to respond more spontaneously in length and depth, which explains the emergence of codes that diverged from those anticipated.

Collectively, these cases likely contributed to the low counts for unrelated themes in Q1 histograms and suggest that, compared to the other questions, Q1 was more susceptible to variation in interview delivery.

6. World event context in relation to narrative theme

As part of the exploratory analysis of this study, we examined broader context factors, particularly the COVID-19 pandemic and its significant disruptions to health care, which were reflected in the TIMS recordings. This intervention was developed early in the pandemic time frame; we anticipated that these shifts in everyday life and medical care delivery would be observable in the interviews. However, an analysis of the recordings from the program’s inception in March 2020 to the completion of this dataset in November 2024 showed no significant presence of these themes in the interviews. A yearly interview (2020–2024) composition from the AI-assisted data analysis was developed, and no observable differences were found in the five main themes identified. Thematic researchers compared the codes found and were able to categorize them into broader categories and determine that the proportionality of appearance was similar regarding the number of interviews assessed for that particular year. There was no consideration of subject composition, however, because these details were not available in the anonymized transcript dataset. The results from this temporal exploratory analysis are observed cautiously as they may not be generalizable beyond this dataset.

## 5. Limitations and Future Directions

This thematic analysis had several limitations related to being conducted at a single site with a large number of interviews from an ICU, physician/chaplain-nominated subjects, surrogate interviewees, AI-assisted code creation/identification and interviewer variability. A major limitation is the corpus of interviews being analyzed as it is a limited set that comes from ICUs and general medical/surgical units, with the majority of them collected during the COVID-19 visitor restrictions and the remaining during the following years as medical institutions revised common hospitalization practices because of the experiences learned from the pandemic. These units either were on biomedical lockdown, with limited to no visitors, or had recently (<1 year) been in lockdown with revised visiting procedures during the collection of many of these interviews. These factors may make the results less generalizable to other patient populations, units, or even institutions.

A large number of these interviews also shared the same interviewer, which may introduce bias into the thematic composition. The approach used by the main interviewer was to ask the questions to the patient or their LAR without them having any prior knowledge of what the questions would be. This person is also a professional medical journalist with decades of experience with productive conversations, assessing reliable responses and obtaining detailed information from people. This, however, may not have fully mitigated the bias factor in the original audio interviews. Consistently, the four questions were asked in a similar order with similar emphasis; however, follow-up questions may have varied.

Another limitation is that transcription and preliminary theme generation involved automated tools, although all outputs were manually reviewed and refined by the coding team. The coding team systematically reviewed transcripts to ensure the generated codes accurately reflected participant data. Specifically, each code was cross-referenced to the corresponding original transcript to verify that it was directly supported by what was mentioned by the participant at that point in the transcript. The potential bias introduced consisted of over-generalizing certain thematic patterns, providing more prominence to common themes over more subtle ones and missing very specific context language, the last being the most probable given the unique hospital setting. All outputs were reviewed by human coders and verified by transcript review and consensus of attribution. In the process of qualitative analysis, it was methodologically legitimate to utilize AI in the initial analysis.

Subjects for the original audio studies were nominated by either a physician or the research chaplain associated with the unit. Due to familiarity with TIMS in the unit, from previous studies, this could have introduced bias in terms of the selection of participants that would have been more able to provide a more complete interview. Consideration for more open subject recruitment was not available during most of this study as a small staff population was used to record these interviews. The potential bias was noted in the analysis.

Lastly, another limitation arises from the use of LARs in the interview process as these responses are not equivalent to patient self-reported themes, as they are a proxy narrative and may have accounted for an external account of the patient’s experience and cannot be fully substituted for the patient’s lived experiences. These proxy responses may differ from the patient self-report narratives and could have introduced interpretive variability into the analysis. Emphasis was not placed on determining whether the transcript was from the self-reported patient narrative or proxy, and this was not accurately accounted for in the captured patient demographics. Emphasis during data collection was placed on age, identified gender and ethnicity for a concordance review to verify, which reflected the composition of the medical unit at the time but not whether it was the patient’s voice or proxy.

Future directions of this intervention include expanding the interviewer’s role to other medical professionals such as nurses and physicians. Further analysis will need to be done to assess the responses to a different type of interviewer for concordance to the observations of this study. Additionally, a mobile application is being developed to prompt, record and, using artificial intelligence, edit the interview to standardize the intervention to reduce interviewer variability while also capturing valuable metadata for future analysis, such as whether the person recording is the patient or a surrogate [7].

## 6. Conclusions

By codifying themes from 362 interviews, twelve main themes were identified, and five main themes appeared with significant frequency. The commonalties of the interview themes were identified in Experience and Quality of Care, Family and Social Connections, Peace and Well-being, Joy and Meaning in Life, and Preferences and Personal Identity.

This thematic analysis suggests that each question in the TIMS interview is associated with eliciting distinct themes that are important for building an understanding of patients outside of what can be understood through medical records. The four questions routinely brought forth recurring and overlapping themes related to personal details, relational support, care-related preferences and meaning-making sources that are not typically found in medical records.

## Data Availability

Thematic codes and representative quotations are available. However, the original transcripts are not available due to ethical considerations of patient privacy. Appendix A provides a minimal dataset to support this paper’s findings.

## Supplementary Materials

The following supporting information can be downloaded at: https://www.mdpi.com/article/10.3390/healthcare14182994/s1, Table S1: Final Codebook; Table S2: Theme-to-Question Mapping; Table S3: Representative Quotations by Theme; Table S4: Interview Variations and Outlier Cases.

## Abbreviations

The following abbreviations are used in this manuscript:

TIMS	This Is My Story
EHR	Electronic Health Record
ICU	Intensive Care Unit

## Appendix A

**Theme**	**Common Code Associations**
Experience and Quality of Care	SATISFACTION_WITH_CARE;
	SATISFACTION_WITH_MEDICAL_TEAM;
	APPRECIATION_OF_MEDICAL_TEAM;
	POSITIVE_INTERACTIONS_WITH_MEDICAL_TEAM; CARE_REQUIREMENTS
Family and Social Connections	FAMILY_BRINGS_JOY; FAMILY_IMPORTANCE; FAMILY_AS_SOURCE_OF_JOY;
	JOY_FROM_CHILDREN;
	SUPPORT_FROM_FAMILY
Peace and Well-Being	SOURCE_OF_PEACE;
	BRINGS_PEACE; PEACE_IN_PRAYER;
	PEACE_INDEPENDENCE;
	PEACE_GOOD_FOOD_MUSIC_CONVERSATION
Joy and Meaning in Life	SOURCE_OF_JOY; BRINGS_JOY;
	FAMILY_JOY;
	JOYFUL_ACTIVITIES;
	HELPING_OTHERS_BRINGS_JOY
Preferences and Personal Identity	PREFERRED_NAME;
	NAME_PREFERENCE;
	PREFERENCE_FOR_NAME;
	PREFERENCES_FOR_ADDRESS;
	PREFERRED_FORM_OF_ADDRESS
Daily Life, Leisure, and Activities	SOURCES_OF_JOY; LOVES_MUSIC;
	JOY_IN_MUSIC;
	ENJOY_READING;
	ENJOY_COOKING
Illness Experience and Coping	DESIRE_FOR_HEALTH;
	EFFECT_OF_MEDICATION;
	DISLIKE_CHANGE_IN_ABILITY; C
	ONCERN_ABOUT_RECOVERY;
	STRUGGLE_WITH_PAIN
Communication and Interaction with Health Care Team	NEED_FOR_COMMUNICATION;
	PREFERENCE_FOR_INFORMATION;
	PREFERENCE_FOR_EXPLANATIONS;
	VALUE_UPFRONT_COMMUNICATION;
	COMMUNICATION_IS_KEY
Meaning, Faith, and Values	FAITH_IN_GOD;
	FAITH_AS_SUPPORT;
	DEVOUT_CATHOLIC;
	PEACE_IN_PRAYER;
	RELATIONSHIP_WITH_GOD
Emotional Experiences and Coping	SOURCES_OF_PEACE;
	ANXIETY_DUE_TO_UNKNOWN;
	ANXIETY_OVER_CHANGE;
	EASILY_ANXIOUS;
	HOPE_FOR_THE_FUTURE
Support Roles and Social Responsibility	HELPING_RELATIVE;
	HELPING_PEOPLE;
	COMMUNITY_IMPORTANCE;
	SUPPORT_IMPORTANCE;
	REALIZATION_OF_SUPPORT
Quality of Life and Well Being	CONCERN_QUALITY_OF_LIFE;
	MINIMIZE_TREATMENT;
	CONCERN_COMMUNICATION_AND_EATING;
	CONCERN_FEEDING_TUBE_DURATION;
	CONCERN_QUALITY_OF_LIFE_POST_SURGERY;
	DESIRE_MINIMAL_INTERVENTION
