# Supplementary material for: This Is My Story (TIMS), a Five-Year Thematic Analysis of Patient Narratives from a Novel Intervention in an Academic Hospital
Source: Healthcare (Basel). 2026 Sep 14;14(18):2994. doi: 10.3390/healthcare14182994 (PMC13606728; doi:10.3390/healthcare14182994)
Supplement: Supplementary file 1 [file healthcare-14-02994-s001.zip › healthcare-4503098-supplementary.pdf]

Supplemental Methods, Codebook, and Theme Mapping for This Is My Story (TIMS): A Five-Year Thematic Analysis of Patient Narratives From a Novel Intervention in an Academic Hospital

Contents

1. Supplementary Table S1. Final Codebook
2. Supplementary Table S2. Theme-to-Question Mapping
3. Supplementary Table S3. Representative Quotations by Theme
4. Supplementary Table S4. Interview Variations and Outlier Cases
5. Closing Notes

Supplementary Table S1. Final Codebook

Theme	Definition	Standardized Codes	Representative Quote
**Experience and Quality of Care**	Patient reflections on the quality of care received, including satisfaction, trust, communication, clinician behavior, teamwork, transparency, and overall hospital experience.	Satisfaction with care; Satisfaction with medical team; Appreciation of medical team; Positive interactions with medical team; Care requirements; Care attentiveness; Care positivity; Desire for effective treatment; Teamwork importance; Medical team doing well; Negative medical experience; Need for clinical overview; Hospital avoidance; Helping nature; Need for positivity; Concern about team conflict; New experience; Finest hospital people; Medical background; Communication importance	"I think they know pretty well. This team down here is the best team, I think, in the hospital."
**Family and Social Connections**	Mentions of family members, friends, caregiving relationships, visits, social support, relational joy, and belonging.	Family brings joy; Family importance; Family as source of joy; Family joy; Joy from family; Joy from family and friends; Joy from friends; Joy from children; Support from family; Support system; Social support role; Family details; Family status; Family information; Family structure; Family location; Family dynamics; Family relationships; Family communication; Recent visit; Family presence; Family taking care; Gratitude for family reunion; Family visits; Close-knit family; Long marriage; Family and friends mentions; Brother's caretaking role; Granddaughter visit; Family activity; Family circumstance; Concern for child; Advice to child; Grandmother support	"What brings me joy is my family, being around my family, enjoying their birthdays, their get-togethers."

Theme	Definition	Standardized Codes	Representative Quote
**Peace and Well-Being**	Statements about peace, calm, comfort, safety, grounding, gratitude, acceptance, and spiritual reassurance.	Source of peace; Brings peace; Peace in prayer; Peace in independence; Peace through food, music, or conversation; Getting out brings peace; Nothing currently brings peace; Outgoing yet quiet; People-oriented; Lack of peace; Peace through calming music or nature sounds; Peace and gratitude; Peaceful moments; Scriptural peace; Personal peace; Peace of mind importance; Finding peace in space; Work brings peace; Family brings peace; Peace when others are happy; Acceptance importance; Gratitude reevaluation; Peace through relaxation; Peace through white noise; Finding peace at home	"God. Jesus. He brings me a lot of peace."
**Joy and Meaning in Life**	Sources of joy, purpose, fulfillment, meaning, satisfaction, and what makes life feel worthwhile.	Source of joy; Brings joy; Family joy; Joyful activities; Helping others brings joy; Finding joy in others' happiness; Things that bring joy; Joy in music; Joy in entertainment; Joy in travel; Joy in water; Joy from family; Joy from family and friends; Joy from friends; Joy from family and cat; Joy from sports and outdoors; Joy from grandchildren's sports; Joy from weekends; Joy from religious practice; Joy from plain living; General happiness; Beautiful life; Phenomenal life; Phenomenal career; Lack of joy; Sources of satisfaction; Meaning in relationships; Purpose in everyday life	"My family, my wife, playing ball, playing softball… Friends, boating."
**Preferences and Personal Identity**	Preferred names, titles, forms of address, self-identification, occupational identity, and identity-related descriptions.	Preferred name; Name preference; Preference for name; Preferences for address; Preferred form of address; Preferred address; Current name; Current name preference; Currently satisfied with address; Dislike being called Mr. / title-related concern; Change in name preference; Identity and relation; Identity confusion; Personality traits; Easygoing nature; Stubbornness; Self-blame; Desire to recover; Desire to return home; Professional background; Occupational identity; Professional history; Previous job; Job loss; Occupation; Employment; Introversion; Private person; Social identity; Social role; Identifies as gamer; Simple personality; Self-awareness	"I prefer to be addressed the right way, you know."

Theme	Definition	Standardized Codes	Representative Quote
**Daily Life, Leisure, and Activities**	Hobbies, routines, recreation, pets, entertainment, travel, reading, cooking, music, and other everyday activities.	Enjoys music; Music preference; Current music playing; Joy in music; Enjoy reading; Enjoy cooking; Enjoy hunting and fishing; Travel and love; Enjoy travel; Joy in travel; Joy in sports and outdoors; Water activities; Gardening; Painting interest; Painting talent; Cooking show idea; Videotaping meals; Music interest; Leisure activities; Outdoor person; Fresh air; Dog ownership; Cat ownership; Pet description; Animal lover; Love for animals; Special Olympics participation; Sports preference; Sports interest; Watching family sports; Outdoor recreation; Reading habits; Favorite TV/show preferences	“A lot of stuff. Music. Movies.”
**Illness Experience and Coping**	Experiences of illness, symptoms, pain, treatment, recovery, adaptation, symptom burden, and coping with medical challenges.	Desire for health; Effect of medication; Concern about recovery; Struggle with pain; Painful experience; Pain need for sleep; Masking pain; Focus on illness not pain; Health knowledge; Healthcare wishes; View on illness; Symptom downplaying; Cooperation with condition; Fear of illness progression; Need for rest; New environment concern; Medical burden; Desire to recover	“I always am like not sure what’s happening.”

Theme	Definition	Standardized Codes	Representative Quote
**Communication and Interaction with Healthcare Team**	Preferences about communication with clinicians, understanding information, being listened to, directness, explanations, and clarity in care conversations.	Need for communication; Preference for information; Preference for explanations; Value upfront communication; Communication is key; Communication difficulty; Preference for directness; Doctors not listening; Communication importance; Importance of involvement; Need for clinical overview; Information seeking; Transparency in care; Preference for direct answers; Communication with spiritual support; In communication with spiritual support; Flexibility in address; Preference for address; Staff should explain clearly; Need for reassurance through communication	"Just working with me and letting me know the situation so that I can fully understand what's happening."
**Meaning, Faith, and Values**	Religious belief, prayer, faith, spiritual practice, moral values, existential meaning, and spiritually grounded coping.	Finds peace in religion; Spirituality importance; Faith in highest being; Faith sustains in difficulty; Belief in Lord's path; Man of God; Faith peace; Strengthening belief; Important to know believer in Jesus Christ; Important to know Christian / important life; Peace in Jesus Christ; Church attendance; Spiritual practices; Religious support; Spiritual backing from family; Religion as source of peace; Spiritual peace; Peace religion; Scriptural peace; Prayer; God-centered coping; Life philosophy; Good heart; Religious values; Meaning through faith; Values-based coping; Christian identity	"I'm a religious person and I talk to God."
**Support Roles and Social Responsibility**	Caregiving roles, responsibilities to others, helping behaviors, and social obligations.	Helping others; Helping others brings joy; Helping others importance; Support roles and social responsibility; Social support role; Granddaughter help; Family caregiver focus; Brother's caretaking role; Grandmother support; Parenting challenges; Wealth and provision; Concern for child; Advice to child; Family support/caregiving; Responsibility to others; Reciprocal support; Caring for spouse/family member	"I like to help people."
**Emotional Experiences and Coping**	Emotional responses, coping strategies, resilience, hope, worry, distress, gratitude, acceptance, and emotional processing.	Hope for the future; Trying to remain positive; Positive thoughts/energy; Move forward; Self-talk and religion; Scary situation; Struggle with control; Concern for health; Thankfulness; Gratitude; Acceptance importance; Emotional experiences and coping; Sources of peace; Peace of mind importance; Anxiety; Fear; Worry; Calmness; Resilience; Coping strategies; Emotional adjustment; Stress management	"I'm trying to remain positive."

Theme	Definition	Standardized Codes	Representative Quote
**Other / Miscellaneous**	Rare, ambiguous, or uncategorized content not confidently assigned to a primary theme.	Miscellaneous; Unclear fit; Rare outlier content; Protocol deviation content; Cross-theme residuals	—

Supplementary Table S2. Theme-to-Question Mapping

Final Theme	Q1: Preferred Address	Q2: Joy	Q3: What the Team Needs to Know	Q4: Peace	Notes on Overlap / Ambiguity
Experience and Quality of Care	Low	Moderate	High	Low	Most strongly associated with Q3, but also appeared across other prompts.
Family and Social Connections	Low	High	Low	Low	Most often linked to Q2; occasional overlap with identity or peace responses.
Peace and Well-Being	Low	Low	Low	High	Strongest association with Q4; often included spiritual or coping language.
Joy and Meaning in Life	Low	High	Low	Low/Moderate	Frequently co-occurred with family and leisure content in Q2.
Preferences and Personal Identity	High	Low	Low/Moderate	Low	Most strongly associated with Q1, but some identity-related content also appeared in Q3.
Daily Life, Leisure, and Activities	Low	High	Low	Low	Common in Q2 responses about hobbies, routines, and recreation.

Final Theme	Q1: Preferred Address	Q2: Joy	Q3: What the Team Needs to Know	Q4: Peace	Notes on Overlap / Ambiguity
Illness Experience and Coping	Low	Low/Moderate	High	Moderate	Most often linked to Q3, though coping language also appeared in Q4.
Communication and Interaction with Healthcare Team	Low	Low	High	Low	Predominantly associated with Q3.
Meaning, Faith, and Values	Low	Low	Low/Moderate	High	Most commonly appeared in Q4.
Support Roles and Social Responsibility	Low	Moderate	Low	Low	Often appeared in Q2 when family or caregiving responsibilities were described.
Emotional Experiences and Coping	Low	Low/Moderate	Moderate	High	Most prominent in Q4, but also appeared in Q3.
Other / Miscellaneous	Variable	Variable	Variable	Variable	Used sparingly for responses not clearly aligned with a single prompt.

Legend: Low / Moderate / High indicates relative frequency of association with each prompt based on coding review.

Supplementary Table S3. Representative Quotations by Theme

Theme	Representative Quote 1	Representative Quote 2	Representative Quote 3
Experience and Quality of Care	"I think they know pretty well. This team down here is the best team, I think, in the hospital."	"Just working with me and letting me know the situation so that I can fully understand what's happening."	"They've been great."
Family and Social Connections	"What brings me joy is my family, being around my family, enjoying their birthdays, their get-togethers."	"My family, my wife, playing ball, playing softball."	"What brings me joy is my family, being around my family."

Theme	Representative Quote 1	Representative Quote 2	Representative Quote 3
Peace and Well-Being	“God. Jesus. He brings me a lot of peace.”	“Music, any kind of music.”	“I listen to it every night before we go to bed, that’s how we go to sleep.”
Joy and Meaning in Life	“A lot of stuff. Music. Movies.”	“My family, my wife, playing ball, playing softball.”	“Everything that we do is family-centric.”
Preferences and Personal Identity	“I prefer to be addressed the right way, you know.”	“Doctor, mister, reverend.”	“Roger. And that’s how you prefer to be addressed, as Roger?”
Daily Life, Leisure, and Activities	“A lot of stuff. Music. Movies.”	“I love music.”	“Boating, which I can’t do anymore.”
Illness Experience and Coping	“I always am like not sure what’s happening.”	“I got terribly cold and started shivering.”	“I had trouble there at first.”
Communication and Interaction with Healthcare Team	“Just working with me and letting me know the situation.”	“They don’t call me nothing.”	“They’ve done well.”
Meaning, Faith, and Values	“I’m a religious person and I talk to God.”	“He brings me a lot of peace.”	“I just called on Jesus.”
Support Roles and Social Responsibility	“I like to help people.”	“We got the bills taken care of.”	“Caring for my family is important.”
Emotional Experiences and Coping	“I’m trying to remain positive.”	“It was scary.”	“I was always worried about her pain.”
Other / Miscellaneous	—	—	—

Supplementary Table S4. Interview Variations and Outlier Cases

Case Type	Description of Variation	Example	Potential Effect on Coding	How Handled in Analysis
Non-standard Q1 wording	Interviewer used an open-ended introduction rather than the standard preferred-address question.	"Mind just introducing yourself for the recording?"	May elicit identity, professional, or family-related content beyond preferred address.	Coded according to transcript content and flagged as a protocol variation.
Questions previewed in advance	Interviewer shared all four questions before the formal interview began.	All four prompts listed up front	May shape response structure and reduce separation between question-specific themes.	Noted as an administration deviation.
Surrogate / LAR response	Interview conducted with a legally authorized representative rather than the patient.	Family members answered on behalf of the patient.	May reflect proxy perspective rather than direct patient self-report.	Included in the corpus and interpreted as a surrogate account.
Additional introductory prompt	Interview included a preliminary question before standardized Q1.	"Who are you and how are you related?"	May produce content that maps to more than one theme.	Reviewed during consensus coding.
Elaborated Q1 response	Participants provided more than just a preferred name or title.	Name plus professional background or personal details.	May generate identity and non-identity codes from the same response.	Coded to all supported themes when appropriate.

Closing Notes

These supplementary materials are provided to enhance transparency regarding the development of the TIMS thematic framework, including the use of AI-assisted preliminary coding, coder consensus refinement, mapping of themes to interview prompts, and handling of arability interview structures. They are intended to support interpretation of the final manuscript and provide readers with a clearer audit trail from transcript to theme.
